# Mlle. Louise Laborde

**Published:** 1911-04-08

**Authors:** 


### OBITUARY.

The death is announced from Beauvais, at the age of eighty-three, of Mlle. Louise Laborde (in religion Sœur Mathilde), who saw service in the care of the wounded during the war of 1870. She had been in charge of the nursing arrangements at the military hospital at Beauvais since 1859, and was decorated with the Legion of Honour in 1886 in recognition of her long hospital service and of her devotion to the sick during the war.

